# Why Firing Rate Distributions Are Important for Understanding Spinal Central Pattern Generators

**DOI:** 10.3389/fnhum.2021.719388

**Published:** 2021-09-03

**Authors:** Henrik Lindén, Rune W. Berg

**Affiliations:** Department of Neuroscience, Faculty of Health and Medical Sciences, University of Copenhagen, Copenhagen, Denmark

**Keywords:** spinal cord, central pattern generation, firing rate distribution, motor control, balanced network, lognormal

## Abstract

Networks in the spinal cord, which are responsible for the generation of rhythmic movements, commonly known as central pattern generators (CPGs), have remained elusive for decades. Although it is well-known that many spinal neurons are rhythmically active, little attention has been given to the distribution of firing rates across the population. Here, we argue that firing rate distributions can provide an important clue to the organization of the CPGs. The data that can be gleaned from the sparse literature indicate a firing rate distribution, which is skewed toward zero with a long tail, akin to a normal distribution on a log-scale, i.e., a “log-normal” distribution. Importantly, such a shape is difficult to unite with the widespread assumption of modules composed of recurrently connected excitatory neurons. Spinal modules with recurrent excitation has the propensity to quickly escalate their firing rate and reach the maximum, hence equalizing the spiking activity across the population. The population distribution of firing rates hence would consist of a narrow peak near the maximum. This is incompatible with experiments, that show wide distributions and a peak close to zero. A way to resolve this puzzle is to include recurrent inhibition internally in each CPG modules. Hence, we investigate the impact of recurrent inhibition in a model and find that the firing rate distributions are closer to the experimentally observed. We therefore propose that recurrent inhibition is a crucial element in motor circuits, and suggest that future models of motor circuits should include recurrent inhibition as a mandatory element.

## 1. Introduction

Although it is known that the core neural elements of rhythmic movement, the central pattern generators (CPGs), are located in the spinal cord and the medulla, the neuronal architecture of these networks has remained perplexing. Several working hypotheses for the principle behind generation of movements have been proposed, e.g., muscle synergy and traveling wave (Cuellar et al., 2009; Saltiel et al., 2016, 2017; Yokoyama et al., 2017), multiple unit burst generators (Grillner, 1981), and multilayered half-center organization CPG (Ivanenko et al., 2006; McCrea and Rybak, 2008). A common theme in the literature is the half-center organization inspired by Brown (1914), where two rhythm generating modules, which have recurrent excitation, are coupled reciprocally via inhibitory populations to ensure an alternating flexor and extensor activity (McLean and Dougherty, 2015; Kiehn, 2016; Grillner and El Manira, 2020). Using optogenetics and light activation or inhibition of spatially restricted regions in the spinal cord it was possible to exclusively activate either flexor or extensor rhythms suggesting a modular organization (Hägglund et al., 2013). A modular organization has also been suggested based on analysis of rodent gaits and cellular ablation (Bellardita and Kiehn, 2015) and gaits of human infants (Sylos-Labini et al., 2020). It has been widely suggested that the rhythm generating modules are composed of a recurrently connected excitatory networks, where the rhythm is generated by a subset of neurons with pacemaker properties (Grillner and El Manira, 2020). Nevertheless, modular structures has been difficult to further isolate experimentally (Auyong et al., 2011a,b), and many circuit elements seem to be involved (McLean et al., 2008; Pham et al., 2020) and therefore the details of their architecture, e.g., whether they indeed are composed of recurrent excitation, have been difficult to substantiate. From a stability point of view, however, it is well-known that a population of recurrently connected excitatory neurons without inhibitory interneurons have a propensity to increase their activity in a catastrophic runaway manner (Hennequin et al., 2017; Berg et al., 2019). So, what decides if a network is stable and would a network composed purely of recurrent excitation be sensible for generation of motor activity?

## 2. Recurrent Networks: Stable or Unstable?

**Figure 1:**
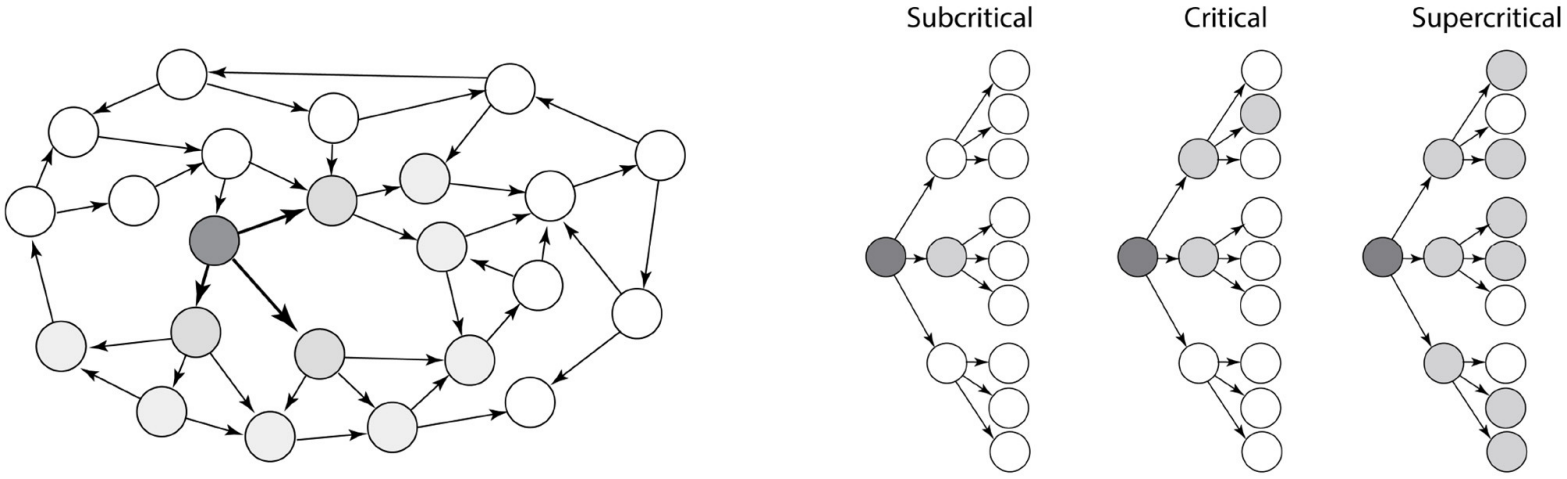
Dynamics of a recurrent excitatory network. **(Left)** A recurrent excitatory network often has embedded tree-like structures, e.g., the dark nodes. **(Right)** The stability of networks with tree-like structure are more simple to characterize. They are either subcritical (left), critical (middle), or supercritical (right). Activity in supercritical networks are unstable and will activate the entire network as exponential growth, whereas the activity in subcritical networks will decay exponentially. Adapted with permission from Larremore et al. (2014).

There are many different types of network structures, and the topology of a network determines its stability. This is especially true for recurrent excitatory networks, since activity can create more activity in reverberation and runaway activity. However, recurrent excitatory networks can also become completely silent. So, what decides the stability of an recurrent excitatory network? We may gain some intuition by viewing the recurrent network as a tree-like structure with feed-forward motifs (Figure 1). The overall topology can be approximated as a tree-like network, also termed a branch–process, which is simpler to understand. This tree-like assumption has been shown to be a good approximation for many types of real recurrent networks (Melnik et al., 2011).

Branching processes was first studied by Galton and Watson without relation to neuroscience, but in regards to extinction of aristocratic families, passing down family names from one generation to the next (Watson and Galton, 1875). Another example of branching processes is nuclear chain reactions. Fission is utilized in nuclear power plants, and this process also has a tree-like structure. Keeping the reaction going requires careful regulation between activity and curbing the fission process.

The activity that propagates in a tree-like network can either be subcritical (left, Figure 1), critical (middle) or supercritical (right). Whether a network is sub-critical or supercritical depends on the so-called the “branching ratio,” i.e., the expected number of action potentials in the receiver population that an action potential induces. If the branching ratio is below 1, the neuronal activity will rapidly die out (subcritical). If the branching ratio is above 1, it will rapidly increase with exponential growth until the whole population is active (supercritical). If the network is critical, i.e., between sub- and supercritical, there is a simple power-law relation between the number of activated neurons, *n*, i.e., the size of an avalanche, and the probability (Beggs and Plenz, 2003; Larremore et al., 2014):

$$\begin{array}{l} p\left(n\right)\propto n^{-3/2} \end{array}$$

For activity to be between silence and runaway activity, i.e., the critical propagation, the branching ratio has to be exactly 1 (Beggs and Plenz, 2003), which makes it unlikely that the network is in a critical state by accident. Rather it likely has some self-organization toward a critical states in order to possess this property (Hesse and Gross, 2014; Rybarsch and Bornholdt, 2014). If there is no such self-organization, the network is expected to be either subcritical (quiescent) or supercritical i.e., activity is rapidly propagating throughout the whole population.

**Figure 2:**
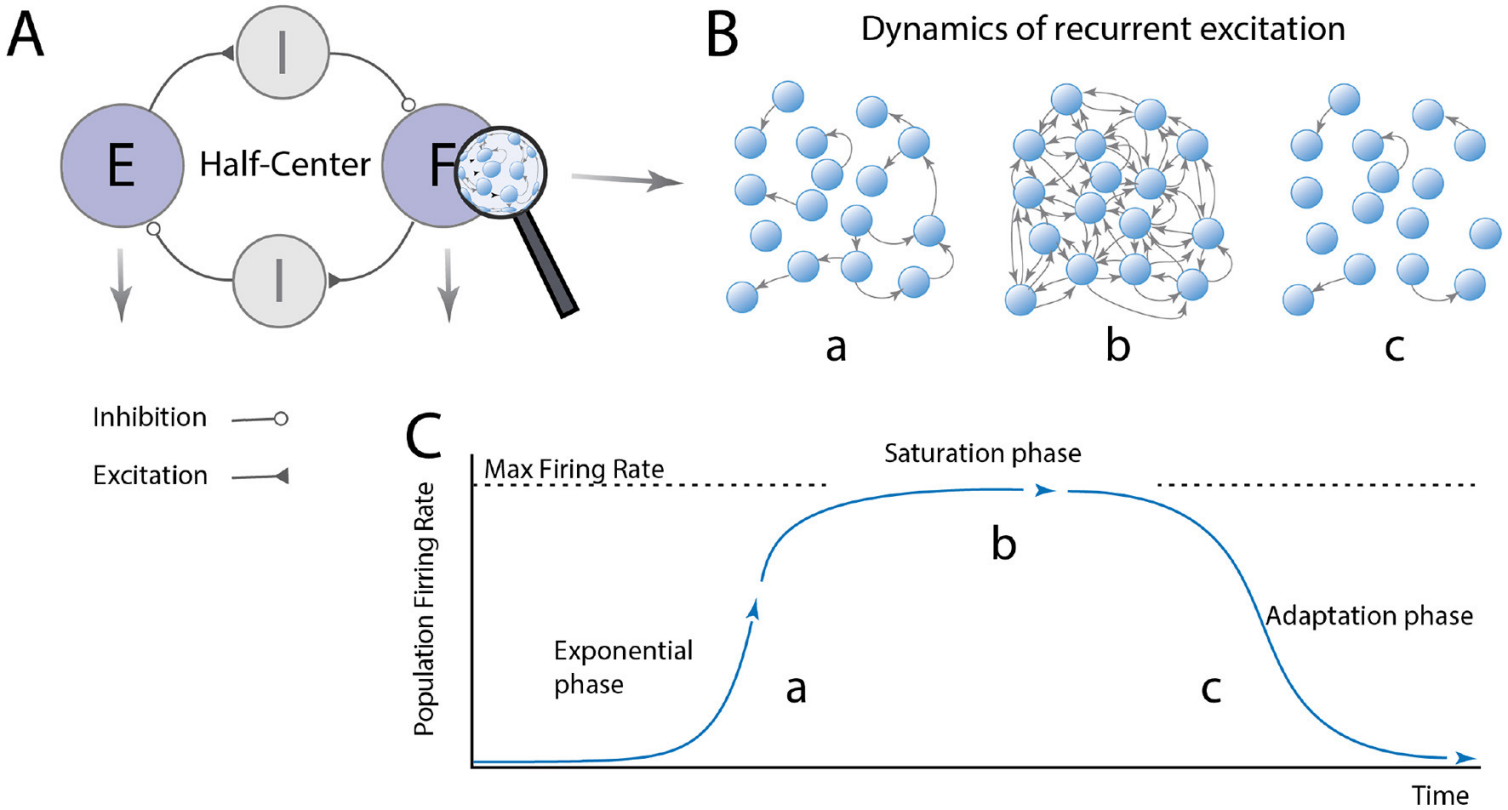
The dynamics of a burst-generator module has 3 phases. **(A)** The half-center organization consists of modules: flexor (F) and extensor (E) modules, which generate rhythmic bursting activity and these are reciprocally connected via inhibitory modules to ensure F/E alternation. **(B,C)** The F/E modules are composed of excitatory neurons with pacemaker properties, which are recurrently connected. The module has three phases: (a) When a neuron fire action potentials (arrows) these quickly activate other neurons, since it is supercritical, resulting in an exponential increase in the network activity. More activity leads to more spikes activating more neurons. Once the activity has propagated to the whole network, the neurons reach their max firing rate the network reach saturation (phase b). Shortly there after the adaptation/pacemaker property turns off the firing in the adaptation phase c, and the activity decay back toward quiescence. During the adaptation phase the quiescence also cease the inhibition to the opposite module, allowing a similar cycle to take place there with a delay. Hence, the population alternates between quiescence and maximal firing rates for all the cells in the module.

So, are CPG modules subcritical or supercritical? Since the neural activity in subcritical networks rapidly dyes out and remain silent, supercritical networks must constitute the basis for rhythm generation in spinal circuits. Spinal motor circuits may involve multiple modules and layers. To keep things simple, let us imagine a simple half-center model that is composed of two supercritical modules with reciprocal inhibition (Figure 2A). When one module becomes active, the network first undergoes a runaway escalation of firing rates where excitation rapidly propagation throughout the network. We consider this “the exponential phase” (Figures 2B,C). Once the whole population is reverberating, the neurons reach their maximum firing rates, and the distribution of firing rates become narrow at the highest possible value. In this saturated phase, adaptation starts to be engaged, e.g., via accumulation of intracellular calcium+, and the firing rates drops back down to zero. After some time, the neurons recover and the cycle can start over. Now we have gained an intuition of the basic principle of recurrent excitatory networks, let us examine how the peak firing rates would be distributed in a network-based model of a half-center module.

## 3. Dynamics of a Recurrent Excitatory Network

**Figure 3:**
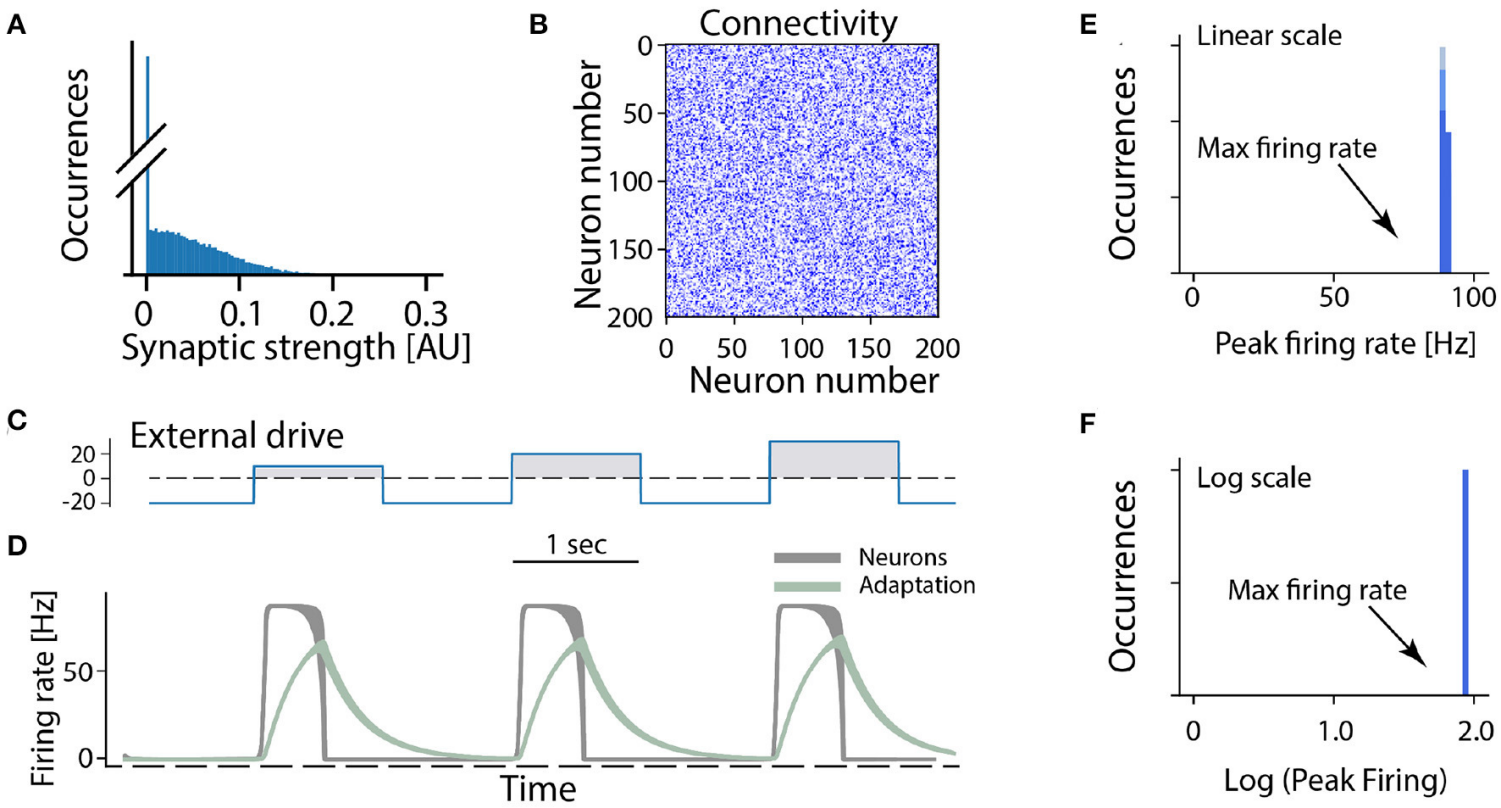
All-or-none behavior of recurrent excitatory networks: model. **(A)** Distribution of synaptic strengths in the model is a truncated Gaussian, with many having zero (no connection). **(B)** Connectivity matrix of the non-zero connections in the network composed of 200 excitatory neurons. **(C)** The external drive to the network has to be kept negative (inhibitory) to keep it silent because the network is unstable (supercritical). Positive pulses was given with increasing amplitude imitating a rhythmic drive. **(D)** The firing rate of the neuronal population (gray overlay) has a prompt increase to maximal firing once the external drive becomes positive. Intrinsic adaptation (green traces) curbs the activity and turns the firing off after some time. Note the population firing is at maximum regardless of input size. **(E,F)** The linear and log distributions of peak firing rates across population have a single mode at maximal firing (arrows) regardless of the input.

In the following we consider a minimal network of recurrently connected excitatory neurons. Let us assume that the excitatory neurons are recurrently connected by synapses which have a wide distribution (and no negative strengths; Figure 3A). The synaptic connection strengths are randomly drawn from this distribution resulting in a connectivity matrix (Figure 3B). If the overall strength of recurrent connections is strong enough the network becomes super-critical, so that a very small amount of spontaneous activity or external activation will result in a burst of neuronal activity. In such a burst all neurons fire at the maximum firing rate in a synchronized manner when provided by different external drives (Figures 3C,D). After a short time intrinsic adaptation in the neurons becomes active to suppress and ultimately terminate the population burst (Figure 3D, gray traces). The adaptation is also the reason that the network response is shorter than the input pulse (cf. Figures 3C,D). An important aspect with this cyclic activity is that the firing rate of the constituent neurons reach their peak firing rates simultaneously, and therefore the distribution of rates across the population is narrow and clustered around the maximum (Figures 3E,F). Hence, if this model is meaningful, it should be relatively easy to verify whether a rhythm—generating module is composed of excitatory recurrent connections by observing the distribution of firing rates across the population and identify those neurons who display a high peak firing rate. Surprisingly, however, the distribution of firing rates have rarely been reported neither in experimental studies nor in theoretical investigations of spinal motor circuits. As we will see next, however, the few experimental observations of firing rate distributions do not seem to be in accord with a high and narrow distribution of firing rates.

## 4. Experimentally Observed Firing Rate Distributions

**Figure 4:**
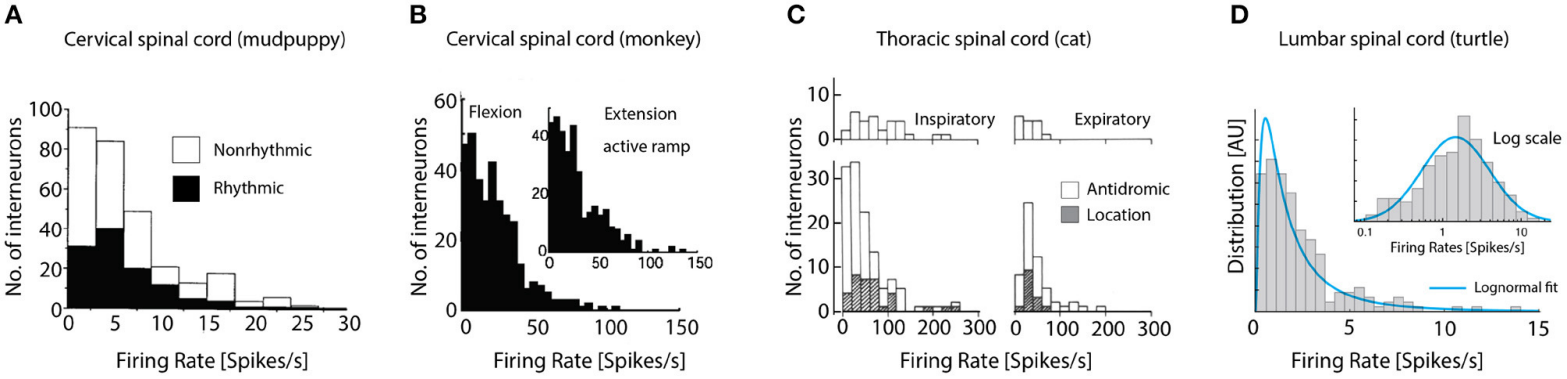
Firing rate distributions across experimental preparations are skewed toward the origin. **(A)** The cervical CPG of the mudpuppy while performing locomotion, show rhythmic (black) and nonrhythmic discharge. **(B)** Cervical interneurons in macaque monkeys performing wrist flexion and extension. **(C)** Interneuronal recording from respiratory motor circuitry in the thoracic cord of cats. Left: inspiratory interneurons, right: expiratory interneurons. Top: identified by antidromic stimulation, bottom inclusion identification by location, white and hatched, respectively. **(D)** Lumbar spinal cord of turtle during scratching has skewed firing rate distribution (left) that is bell-shaped on log scale (blue fit). Reproduced with permission **(A)** Cheng et al. (2002), **(B)** Prut and Perlmutter (2003), **(C)** Kirkwood et al. (1988), and **(D)** Petersen and Berg (2016).

Our survey of the literature provides two observations: First, a distribution of firing rates across neuronal population has rarely been reported (Petersen and Berg, 2016; Cuellar et al., 2018). Second, those experimental reports that do provide the firing rate distribution, they seem in impressive agreement. The precise firing rates vary from experiment to experiment and region to region, but their distributions all seem to have the same long-tail and skewness toward zero. Spinal interneuronal recordings from cervical CPG of the mudpuppy (Figure 4A), show rhythmic (black) and nonrhythmic discharging units during locomotion, both types have distributions lopsided toward the origin. Recordings from the spinal cord in awake macaque monkeys, which were trained to perform visually guided flexion and extension of their wrist in an active ramp provided similar insight (Figure 4B). During both flexion (left) and extension (inset) the distribution of firing rates of interneurons in the cervical spine had a strong skewness toward origin qualitatively similar to the mudpuppy data, in spite of their differences in species and motor function. Interneuronal recording from respiratory motor circuitry in the thoracic spinal cord of cats also provided insight to firing rate distributions in motor circuitry (Figure 4C). Left column: inspiratory interneurons, right column: expiratory interneurons. Top: identified by antidromic stimulation, bottom both antidromic and by location, white and hatched, respectively. Last, recordings from the lumbar spinal cord of turtle during scratching had skewed firing rate distribution, that closely resembles a log-normal distribution (blue fit, Figure 4D), i.e., normally distributed on a log-scale (inset). It is unknown whether the exact shape of the distribution is best described by a log-normal distribution or other skewed distributions, e.g., the gamma-distribution.

**Figure 5:**
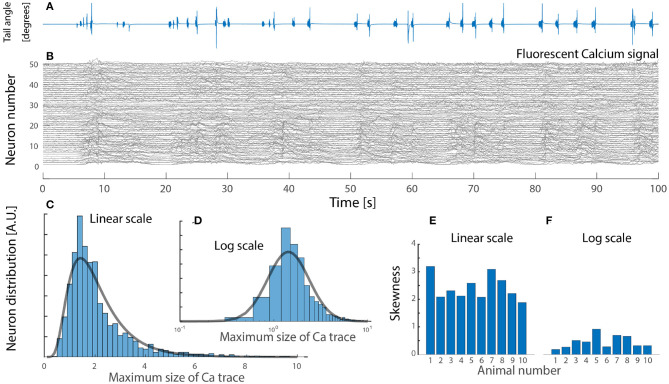
Neuronal imaging of calcium-dependent fluorescent signal in zebrafish larvae hindbrain during visuomotor response has a skewed lognormal-like distribution in peak signal. **(A)** *In-vivo* tail-bending in response to visual input. **(B)** Fluorescent signal (Δ*F*/*F*) at neuronal resolution of a glycinergic subpopulation which provide inhibitory feedback during escape response (50 cells shown). **(C)** The maximal calcium response across cells in the population is skewed and lognormal-like (lognormal fit, gray line). **(D)** Same distribution on log scale resembles a normal distribution (gray fit). **(E)** High skewness across the cohort (*n* = 10 animals). **(F)** Skewness on a log scale is closer to zero, i.e., normal distribution. Data kindly provided by Severi et al. (2018) and reanalyzed with permission.

Due to the rareness of experimental reports, we include and analyze a data set from zebrafish larvae during active locomotion in response to visual input, that was kindly made available (Severi et al., 2018). It is known that inhibitory interneuron V1 and V2b provide feedback inhibition (Callahan et al., 2019; Sengupta et al., 2021) most notably via Renshaw cells. In this preparation it is possible to record the fluorescent signal from a genetically encoded calcium sensor (GCaMP5G), which is expressed in glycinergic interneurons by the domain for expression of the transcription factor “engrailed1b,” which is highly correlated with the onset of locomotion. The fluorescent signal in these inhibitory neurons is an indication of the degree of increase in spiking activity. Hence, using the maximal fluorescent signal across the population, we found the distribution to be skewed, remarkably close to a log-normal (Figure 5). The distribution of fluorescent signal could be interpreted in various ways. First, the activity across the cells is uniform, but the distribution of soma size—hence the fluorescent signal—could be log-normal, although the cells have equal firing rates. Second, the cell size if normally distributed, but their firing rates are log-normally distribution, hence giving a fluorescent signal with a skewed distribution. The increase in fluorescent signal may not ad linearly, as the spiking activity increases. Nevertheless, the observation of a skewed distribution (Figure 5) appear in accord with the previous observation and the most parsimonious explanation is that the neurons has a log-normal distribution of firing rates (blue fit, Figure 4).

In summary, the meta-analysis indicates a profile of activity across the population, which is skewed toward zero, with a long tail toward higher firing rates. This is at odds with the prediction from a recurrent excitatory network as a driver, where the firing rates should cluster around the maximal firing rate and zero. What could explain this apparent lack of congruence between the conventional model and experimental data?

## 5. Could Reciprocal Inhibition Help?

One of the cornerstones of the half-center organization is reciprocal inhibition. Could reciprocal inhibition help stabilize the firing rates so the population does not switch between silence and maximal firing? The short answer is “no.” Reciprocal inhibition exerts its effect on the antagonist module, which is either already silent, or will become silent once the reciprocal inhibition performs its action. When the activity of the antagonistic module is reduced or completely silent, the returning reciprocal inhibition is also reduced or silent. Hence, reciprocal inhibition actually causes mutual dis-inhibition, which is, in effect, positive feedback. Thus, the reciprocal inhibition in a half-center organization actually provides no help in dampening the firing rates, but rather makes the situation worse, by removing inhibition when it is needed.

## 6. Inclusion of Recurrent Inhibition

**Figure 6:**
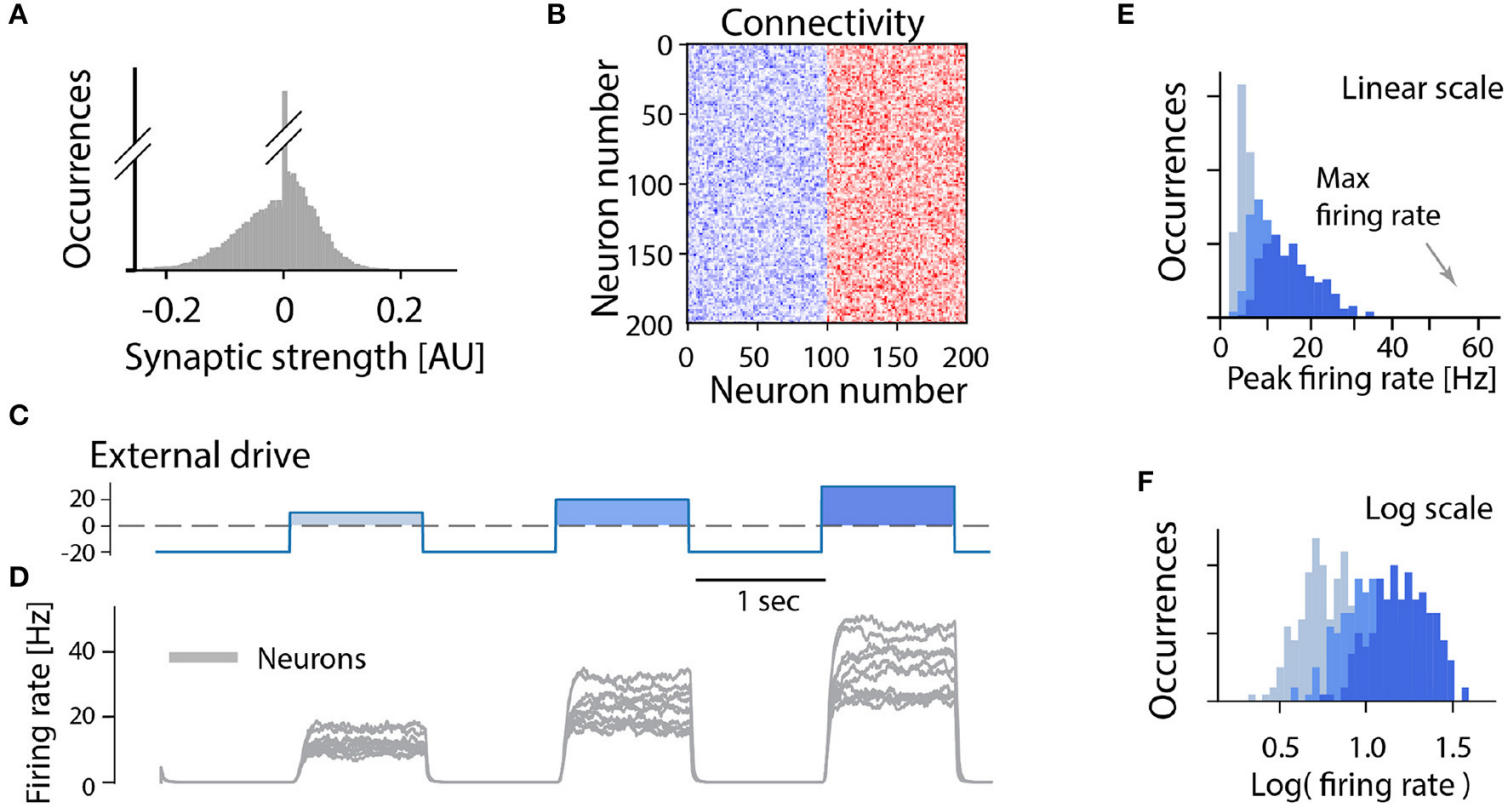
Graded activity in a recurrent excitatory network balanced by inhibition: model. **(A)** Distribution of synaptic strengths in the model is a Gaussian, with a balance around zero. It also have many connections with zero strength (no connection). **(B)** Connectivity matrix of the non-zero connections in the network composed of 100 excitatory (blue) and 100 inhibitory (red) randomly connected neurons. **(C)** The external drive is negative (inhibitory) with positive pulses imitating a rhythmogenic input with increasing amplitude. **(D)** the firing rate of a subset of the neuronal population has diverse peak firing. **(E,F)** The linear- and log-scale distributions of peak firing rates are broad and skewed toward zero far from the maximal firing (arrow). The colors represent increasing external input, light blue: lowest, blue: middle, dark blue: largest input.

**Figure 7:**
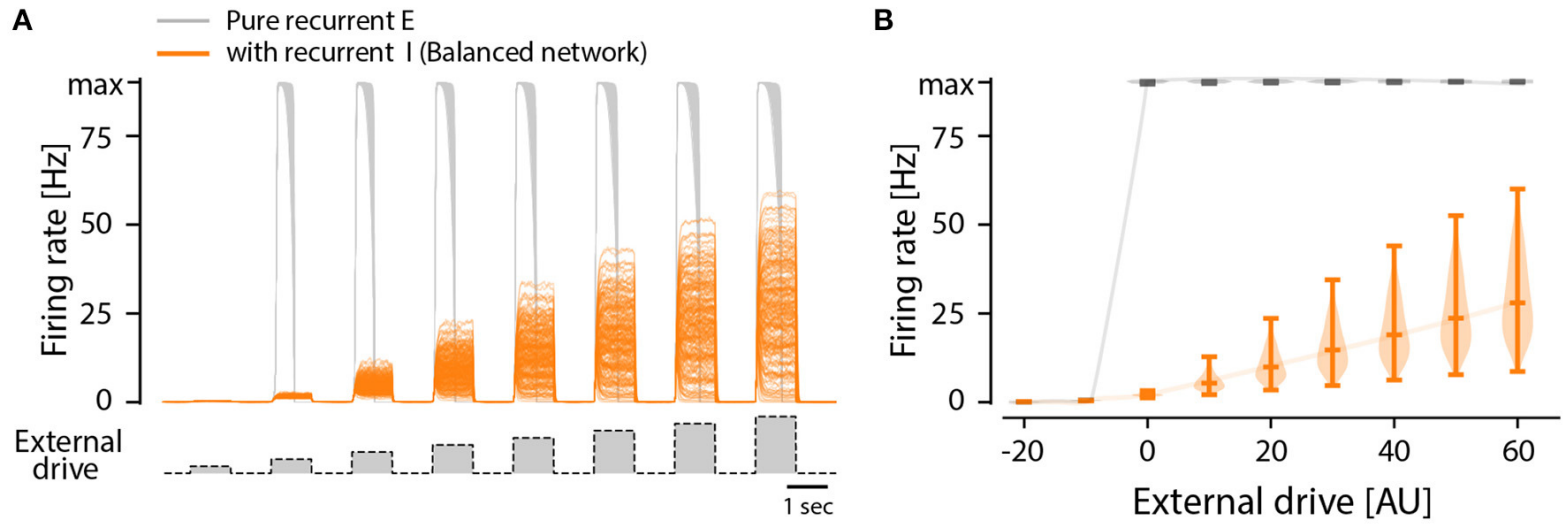
All-or-none vs. graded population response to external drive in two types of model networks: Purely recurrent excitatory network and one including recurrent inhibition, i.e., a balanced network. **(A)** Firing rates of individual neurons in a recurrent excitatory network (gray) and a recurrent network with both excitation and inhibition (orange), i.e., a balanced network, in response to external drive (bottom trace). All neurons in the excitatory network has the same firing rate, i.e., the maximum or minimum, in response to input, whereas the balanced net has a graded mean response with a wide distribution. **(B)** The distribution of firing rates across the population in the balanced net (orange) is wide and has a graded response to input, whereas the excitatory network has an all-or-none response that is narrow.

Although there are indications of a sparse connectivity in the CPG structure (Carroll and Ramirez, 2013; Radosevic et al., 2019) the architecture and connectomics of circuits generating motor programs are largely unknown. We know that reciprocal inhibition is indirectly enhancing the instability via dis-inhibition and therefore it does not provide a solution to the problem. So, what is the simplest means to remedy the divergence between these states? As it turns out, recurrent inhibition has an important element in stabilizing the spinal activity. It is known that firing rate distributions are broad for recurrent networks with a balanced between inhibition and excitation (Vogels et al., 2005; Hennequin et al., 2017) and strongly skewed (van Vreeswijk and Sompolinsky, 1996) as seen in experiments. Recurrent inhibition pulls the membrane potential to be less depolarized and in this way the neuron will spike at a lower rate. This also has the effect that more neurons fire action potentials in the fluctuation–driven regime rather than in the mean–driven regime. It is known that at least half of the neurons are active in the fluctuation driven regime during rhythmic scratching (Petersen and Berg, 2016; Berg, 2017). At the same time balanced inhibition and excitation has been observed in at least a subset of neurons in various motor networks (Berg et al., 2007; Petersen et al., 2014; Vestergaard and Berg, 2015; Ramirez and Baertsch, 2018). To illuminate this further, we expanded the model of a recurrent excitatory network presented earlier (Figure 3) to include recurrent inhibition, which is also known as “a balanced network.” While the pure excitatory network had an all-or-none firing activity, the inclusion of recurrent inhibition to replace the role of intrinsic adaptation enriches the network with the capacity to have a graded response to an external drive (Figure 6). Furthermore, the firing rate distribution turns from having a single mode at the maximal firing rate (Figures 3E,F) to having a widely distributed peak firing across the population (Figures 6E,F). The distributions is also similar to those observed in experiments, i.e., skewed toward zeros with a log-normal appearance. The difference between recurrent excitatory networks and networks where recurrent inhibition is included can be summarized as: (1) the firing rate distributions are very different both in width an location of peaks. (2) the all-or-none vs. graded response to external drive (Figure 7).

## 7. Blocking Inhibition Makes the Network Unstable

If recurrent inhibition is indeed a vital element of motor networks, the effect of reducing inhibition should be to increase the overall firing rates and to synchronize the spinal network. Several experiments have been performed where the effects of a systemic block of inhibition by application of either glycinergic antagonists (e.g., strychnine) or GABAergic antagonists (e.g., picrotoxin, bicuculline; Cowley and Schmidt, 1995; Beato and Nistri, 1999; Talpalar et al., 2011, 2013). The general effect is a widespread barrage of intense activity across multiple segments of the spinal cord, which was recorded via the motor nerve output. When reducing inhibition the effective branching ratio grows dramatically, i.e., the expected number of post-synaptic action potential that a presynaptic neuron will increase, and hence the network becomes highly supercritical (Figure 1). A seizure-like barrage of activity quickly spread throughout within milliseconds and reverberates the spinal network until some form of adaptation or fatigue turns it off. This is similar to the dynamics of a recurrent excitatory network (Figure 3). The activity slowly alternates between intense activity with maximal firing of individual neurons and quiescence, which represents a recovery phase.

## 8. Discussion

In spite of the striking insight into the CPG architecture that firing rates distributions can provide, they have received remarkably little attention in the motor control literature. Other parts of neuroscience have identified distributions as an important element in theoretical neuroscience (Vegué and Roxin, 2019) and e.g., in the processing of sleep regulation (Levenstein et al., 2017) and decision making (Wohrer et al., 2013). The idea of the “log-normal” brain is inspired by the skewed shape of the firing rate distributions (Mizuseki and Buzsáki, 2013; Buzsáki and Mizuseki, 2014).

Here, we inspect the dynamics of excitatory recurrent networks driven by an external drive. Despite the absence of experimental evidence, this type of network architecture has often been proposed to constitute a module in a half-center model or unit burst generator (McCrea and Rybak, 2008; Grillner and El Manira, 2020). Nevertheless, for a recurrent network to not be silent, it has to be supercritical. In such networks, propagation of activity is rapid due to the tree-like structure (Figure 1), which makes it unstable and prone to acceleration and reverberation until all cells fire at maximal rates. This also causes recurrent excitatory networks to essentially work by all-or-none activity: Either it is silent or it has maximal activity (Figure 7). A consequence of this is that it does not respond in a graded fashion to various external drives and has no ability to modulate the output amplitude. This lack of flexibility seems sub-optimal given the large variety of motor task that the body needs to execute, e.g. fine motor skills vs. heavy lifting, and walking peacefully vs. carrying a large load or walking uphill. Hence there is a strong functional argument against an modular architecture of pure recurrent excitatory connectivity.

Furthermore, comparing the firing rate distributions of recurrent excitatory networks with those from experimental reports, they are incompatible: Experiments show broad distributions skewed toward very low rates, akin to a log-normal (Figures 4, 5), whereas recurrent excitatory networks have a single mode at maximal firing, or at zero (Figures 3E,F). A single mode at maximal firing is the exact opposite of the experimentally observed low firing with a wide skewed distribution. Hence, there are functional arguments against the hypothesis of a recurrent excitatory network as well as experimental evidence against it.

So, if there are both experimental and functional arguments against recurrent excitatory modules, what type of network architecture could constitute modules? As the most parsimonious solution, we suggest including recurrent inhibition to curb the activity. Networks that encompass both recurrent excitation and inhibition has previously been considered “heterodox” and “anarchistic” (Grillner and Jessell, 2009), but so far there are no clear experimental evidence against the presence of recurrent inhibition in motor networks. On the contrary, indications of concurrent inhibition have been observed in various medullary and spinal experiments (Ramirez and Baertsch, 2018; Berg et al., 2019). Such balanced networks are also known to have skewed firing rate distributions with low mean firing (van Vreeswijk and Sompolinsky, 1996; Petersen and Berg, 2016). Further, recurrent inhibition is certainly provided via a type of inhibitory interneuron, the Renshaw cell, which receives collateral excitatory fibers from alpha motor neurons, which it also inhibits. Although, the role of these cells is unknown, they may represent just one example of a general and widespread element of recurrent inhibition in spinal motor circuits. Due to the close proximity of Renshaw cells to motor neurons they have been easy to identify, and therefore well-described. Other types of recurrent inhibition in motor network may be less easily identified and therefore likely to receive less attention. There are many other types of inhibitory interneurons (Bikoff et al., 2016), that have remained uncharacterized, because their circuit motifs are more synaptic layers removed from motoneurons.

To test the impact of recurrent inhibition in our simple model, we substituted half of the excitatory neurons in our model with inhibitory neurons, and this changes the dynamics qualitatively in two ways (Figure 6). First, the population response to a different external drive resulted in a graded output (Figure 7), hence allowing a more functional dynamics. Second, the firing rate distribution was wide and skewed toward zero in accord with experimental observations (Figures 6E,F). It is remarkable that such a simple alteration of recurrent excitatory network can change the population dynamics to something more functional and closer to the experimentally observed. Hence, we propose that recurrent inhibition is an indispensable element in motor networks.

## 9. Methods

To understand the effect of recurrent connectivity of the firing rate distribution of spinal networks we consider a population of *N*=200 neurons that we model in terms of their firing rates. The firing rate *r*_*i*_ of an example neuron *i* is determined by a static non-linear firing rate function ϕ(*x*) that relates that activity variable *x*_*i*_ (analogous to a membrane potential) to the output firing rate: *r*_*i*_(*t*) = ϕ[*x*_*i*_(*t*)]. We used

(1) $$\begin{array}{l} \phi \left(x_{i}\right)=\left\{ \begin{array}{cc} r_{0}\left(1+\text{tanh}\left[\left(x_{i}-r_{0}\right)/r_{0}\right]\right), & \text{for }x_{i}\leq r_{0}\\ r_{0}+r_{\text{max}}\text{tanh}\left[\left(x_{i}-r_{0}\right)/r_{\text{max}}\right], & \text{for }x_{i}>r_{0} \end{array}\right. \end{array}$$

where *r*_0_ represents the rate at the inflection point and *r*_max_ = 80 is the maximum deviation of the firing rate from this point. The dynamics of the network is given by

(2) $$\begin{array}{l} \left\{ \begin{array}{c} \tau_{m}{ẋ}_{i}\left(t\right)=-x_{i}+\sum_{j}J_{ij}\phi \left[x_{j}\left(t\right)\right]-g_{w}w_{i}\left(t\right)+I_{e}\left(t\right)\\ \tau_{a}{ẇ}_{i}\left(t\right)=-w_{i}+\phi \left[x_{i}\left(t\right)\right] \end{array}\right. \end{array}$$

where *J*_*ij*_ represents the recurrent connections in the network (see below) and *I*_*e*_(*t*) is a time-varying external drive. The variable *w*_*i*_ represents an intrinsic adaptation that depends on the firing rate and contributes as a negative input to the activity variable *x*_*i*_ with a strength set by the parameter *g*_*w*_. The time constants τ_*m*_ and τ_*a*_ determines the time scale of the activity variable *x*_*i*_ and adaptation *w*_*i*_, respectively. Here we set τ_*m*_ = 50 ms and τ_*a*_ = 300 ms. Below we outline two different scenarios used in this study, either a network with recurrent excitation and intrinsic adaptation (Figure 3) or a network with recurrent excitation and inhibition (but lacking intrinsic adaptation) (Figure 6).

### Recurrent Excitation and Intrinsic Adaptation

For the case of a network with only recurrent excitation that is counteracted by an intrinsic adaptation, we first generate connection weights *J*_*ij*_ from Gaussian distribution with zero mean and a width (standard deviation) set to σ = 0.05. Negative weights are then set to zero, resulting in a truncated distribution with positive only values, where approximately half of the possible connections are zero (Figure 3A). We set the strength of adaptation *g*_*w*_ to match the average sum of the incoming connection weights to each neuron: $g_{w}=1/N\sum_{ij}J_{ij}$.

### Recurrent Excitation and Inhibition

In the model with recurrent excitation and inhibition we first generate the connections as for the case above. Here, however, we consider half of the neurons to be excitatory and half of them to be inhibitory and adjust all connections from the inhibitory connections by a factor *g*_*i*_=-1.5. This results in a network where recurrent connections are dominated by inhibition (Figure 6A). For simplicity we exclude the effect of the intrinsic adaption, i.e., we set *g*_*w*_ = 0.

### Software and Code Availability

Numerical simulations of the network model were done using the forward Euler method implemented in custom-written software using Python 3.8. Code for reproducing Figures 3, 6, 7 is available at Berg Lab website (https://berg-lab.net).

## Data Availability Statement

The datasets presented in this study can be found in online repositories. The names of the repository/repositories and accession number(s) can be found at: https://www.berg-lab.net.

## Author Contributions

Both authors listed have made a substantial, direct and intellectual contribution to the work, and approved it for publication.

## Conflict of Interest

The authors declare that the research was conducted in the absence of any commercial or financial relationships that could be construed as a potential conflict of interest.

## Publisher's Note

All claims expressed in this article are solely those of the authors and do not necessarily represent those of their affiliated organizations, or those of the publisher, the editors and the reviewers. Any product that may be evaluated in this article, or claim that may be made by its manufacturer, is not guaranteed or endorsed by the publisher.
